# Infant Feeding Alters the Longitudinal Impact of Birth Mode on the Development of the Gut Microbiota in the First Year of Life

**DOI:** 10.3389/fmicb.2021.642197

**Published:** 2021-04-07

**Authors:** Modupe O. Coker, Hannah E. Laue, Anne G. Hoen, Margaret Hilliard, Erika Dade, Zhigang Li, Thomas Palys, Hilary G. Morrison, Emily Baker, Margaret R. Karagas, Juliette C. Madan

**Affiliations:** ^1^Department of Epidemiology, The Geisel School of Medicine at Dartmouth, Hanover, NH, United States; ^2^School of Dental Medicine, School of Public Health at Rutgers, Newark, NJ, United States; ^3^Center for Molecular Epidemiology, The Geisel School of Medicine at Dartmouth, Hanover, NH, United States; ^4^Children’s Environmental Health & Disease Prevention Research Center at Dartmouth, Hanover, NH, United States; ^5^Department of Biostatistics, University of Florida, Gainsville, FL, United States; ^6^Marine Biological Laboratory, Josephine Bay Paul Center, Woods Hole, MA, United States; ^7^Department of Pediatrics, Children’s Hospital at Dartmouth, Lebanon, NH, United States

**Keywords:** infant feeding, delivery mode, cesarean section, gut microbiota, infants, breast feeding

## Abstract

Cesarean-delivered (CD) infants harbor a distinct gut microbiome from vaginally delivered (VD) infants, however, during infancy, the most important driver of infant gut microbial colonization is infant feeding. Earlier studies have shown that breastfeeding is associated with higher levels of health-promoting bacteria such and *Bifidobacterium* and *Bacteroides* via modulation of the immune system, and production of metabolites. As the infant gut matures and solid foods are introduced, it is unclear whether longer duration of breast feeding restore loss of beneficial taxa within the intestinal microbiota of operatively delivered infants. Within the New Hampshire Birth Cohort Study, we evaluated the longitudinal effect of delivery mode and infant feeding on the taxonomic composition and functional capacity of developing gut microbiota in the First year of life. Microbiota of 500 stool samples collected between 6 weeks and 12 months of age (from 229 infants) were characterized by 16S ribosomal RNA sequencing. Shotgun metagenomic sequencing was also performed on 350 samples collected at either 6 weeks or 12 months of age. Among infant participants, 28% were cesarean-delivered (CD) infants and most (95%) initiated breastfeeding within the first six months of life, with 26% exclusively breastfed and 69% mixed-fed (breast milk and formula), in addition to complementary foods by age 1. Alpha (within-sample) diversity was significantly lower in CD infants compared to vaginally delivered (VD) infants (*P* < 0.05) throughout the study period. Bacterial community composition clustering by both delivery mode and feeding duration at 1 year of age revealed that CD infants who were breast fed for < 6 months were more dissimilar to VD infants than CD infants who breast fed for ≥ 6 months. We observed that breastfeeding modified the longitudinal impact of delivery mode on the taxonomic composition of the microbiota by 1 year of age, with an observed increase in abundance of *Bacteroides fragilis* and *Lactobacillus* with longer duration of breastfeeding among CD infants while there was an increase in *Faecalibacterium* for VD infants. Our findings confirm that duration of breastfeeding plays a critical role in restoring a health-promoting microbiome, call for further investigations regarding the association between breast milk exposure and health outcomes in early life.

## Introduction

There is overwhelming evidence that the infant gut microbiome plays a fundamental role in regulating both innate and adaptive immune training in early infancy (Cho and Blaser, 2012; de Muinck and Trosvik, 2018). The co-evolution of the microbiota and immune system in infant development represents the hallmark of homeostatic balance between active response to pathogens and tolerance to harmless and beneficial antigens (Coker and Madan, 2020). This time period during which the microbiome is being established, typically the first years of life, coincides with the critical window during which children develop immune-related disorders. Furthermore, infant intestinal microbial dysbiosis has been associated with poor health outcomes such as allergy, atopy and chronic inflammatory disorders later in life (Bisgaard et al., 2011; Abrahamsson et al., 2012; Fujimura and Lynch, 2015). As a result, there is need for a thorough understanding of how the major early-life exposures impact the temporal development of the microbiome from infancy to childhood, particularly in light of promoting preventive and therapeutic interventions.

Several studies examining the effects of early life exposures - including cesarean delivery (Adlerberth, 2008; Dominguez-Bello et al., 2010; Rutayisire et al., 2016; Shao et al., 2019), formula supplementation (Penders et al., 2006; Madan et al., 2016; Hill et al., 2017; Liu et al., 2019; Yang et al., 2019), and intrapartum antibiotic exposure (Aloisio et al., 2014; Zeissig and Blumberg, 2014; Langdon et al., 2016; Stearns et al., 2017; Coker et al., 2019) - have described shifts in overall microbial composition and differential abundance of several keystone bacterial taxa. Studies of 6-week to 6-month old infants show a decrease in abundance of *Bacteroidetes* over time in infants born via cesarean, while *Bifidobacterium* was observed as the most dominant genus in the vaginally delivered group (Yang et al., 2019). These findings have motivated the investigation of microbiota-altering interventions such as vaginal microbial transfer following operative delivery (Dominguez-Bello et al., 2016); however, the long-term impacts and safety of such interventions are unknown. Clarifying how ongoing exposures like infant feeding and antibiotics can modify the long-term influence of delivery mode on the developing gut microbiome has important implications for early life immune modulation and interventions. A few studies suggest that breastfeeding has the potential to restore the gut microbiota of operatively delivered infants to resemble vaginal-born breast-fed infants within first 3 months of life (Hill et al., 2017; Liu et al., 2019). However, larger, prospective studies are lacking.

We and others have previously found that, in neonates, microbiome composition is significantly associated with both delivery mode and feeding mode (Penders et al., 2006; Madan et al., 2016; Hill et al., 2017; Yang et al., 2019). In this study, however, we evaluated combined and independent temporal effects of delivery mode and infant feeding on the taxonomic composition and functional capacity of developing gut microbiota, to determine whether breast feeding can partially restore the infant gut microbiome composition following perturbation from cesarean delivery.

## Materials and Methods

### New Hampshire Birth Cohort Study

The New Hampshire Birth Cohort Study (NHBCS) is an ongoing prospective cohort study of pregnant women and their offspring in rural New Hampshire. Cohort recruitment and data collection procedures have been previously described (Madan et al., 2016; Hoen et al., 2018; Coker et al., 2019). Stool samples used in this study were collected periodically from full-term infants, born at ≥37 weeks gestational age, and with appropriate growth for gestational age.

The study groups for comparison were defined by delivery mode, vaginally delivered (VD) or cesarean -delivered (CD), and feeding from birth up to the time of sample collection (form of infant feeding in the first 6 weeks or duration of breast feeding at 1 year). Children were defined as exclusively breast fed (EBF) if at the time of sample collection, they had not received formula for more than a week since birth and were currently being breast-fed. Based on our previous work (Madan et al., 2016), we defined formula-fed (FF) infants as those who were exclusively formula-fed or breast fed with formula supplementation, i.e., did not meet the criteria for EBF. Duration of breast feeding was measured as time between first exposure to breast milk and time of cessation or sample collection (if still breastfeeding). For 6-week samples, four distinct groups were created for comparison; (i) vaginally delivered and EBF (VD-EBF), (ii) vaginally delivered and FF (VD-FF), (iii) cesarean-delivered and EBF (CD-EBF), and (iv) cesarean-delivered who were FF (CD-FF). Similar to 6-week samples, 1-year samples for those who *ever breastfed* were classified to four groups; (i) vaginally delivered who breast-fed for ≥6 months (VD- ≥ 6BF), (ii) vaginally delivered who breast-fed for <6 months (VD- < 6BF), (iii) cesarean -delivered who breast-fed for ≥6 months (CD- ≥ 6BF), and (iv) CD who breast-fed for <6 months (CD- < 6BF). Six months was chosen as the threshold/cutoff for exclusive breast feeding duration prior to the introduction of complementary foods per recommendations from American Academy of Pediatrics regarding exclusive breastfeeding for 6 months (Kramer and Kakuma, 2004). Additional details regarding feeding classification at time of sample collection can be found in Supplementary Methods.

### Sample Collection, DNA Extraction, and Sequencing

Study participants provided infant stool samples in diapers sealed in separate polyethylene bags and frozen in a home freezer until transport. Samples were transported in a cooler with ice packs and brought in within 24 h of collection to a post-partum visit (at 6 weeks, 4 months, 6 months, 9 months, or 1 year postpartum). These stool samples were frozen at −80°C within 24 h of receipt. Samples were thawed and DNA was extracted using the Zymo DNA extraction kit (Zymo Research).

16S rRNA gene sequencing was performed for all available samples while whole genome sequencing (WGS) was performed on a subset of 6-week and 1-year samples only. 16S amplicons spanned the eubacterial v4 and v5 regions using primers 518F (5′ CCAGCAGCYGCGGTAAN) and 926R (combination of 5′-CCGTCAATTCNTTTRAGT, 5′-CCGTCAATTTCTTTGAGT, and 5′-CCGTCTATTCCTTTGANT). Amplicons are approximately 550 bp with the addition of the fusion primers containing Illumina-specific adapters. Primer numbering is relative to the *E*. *coli* 16S rRNA gene sequence. All samples were prepared for sequencing on the Illumina MiSeq platform using 250 nt paired end reads (16S) or NextSeq platform (shotgun metagenomics) using 150 nt paired end reads at the Marine Biological Laboratory (MBL) in Woods Hole, MA using established methods and as previously published (Huse et al., 2014; Newton et al., 2015; Madan et al., 2016; Hoen et al., 2018; Coker et al., 2019).

### Bioinformatic and Statistical Analysis

Fastq files containing 16S and WGS sequence reads were quality-filtered and trimmed with Kneaddata^1^ and Trimmomatic (Bolger et al., 2014), respectively. 16S rRNA gene sequence processing and analyses were done using R version 3.5^2^. DADA2 sequence processing pipeline (v.1.6.0) (Callahan et al., 2016) was used to infer the amplicon sequence variants (ASVs) present and their relative abundances across samples. Thereafter, taxonomic assignment was performed on ASVs using the Greengenes classifier (DeSantis et al., 2006). We constructed a phylogenetic tree using the DECIPHER (version) and phangorn (v.2.4.0), R packages. All downstream analyses were stratified by delivery mode and based on the eight groups previously described for 6-week and 1-year samples (four groups each).

Within phyloseq (version) (McMurdie and Holmes, 2013), ASV abundances were used to calculate alpha diversity indices. To determine statistical significance of the difference in alpha diversity indices between groups, *F*-tests, Student’s *t*-tests and multivariable linear regression analyses were performed (adjusted for gestational age, antibiotic use and solid food introduction). Using a midpoint-rooted phylogenetic tree generated from phangorn (v.2.4.0), overall community differences between samples (beta diversity) were tested within vegan (v.2.4.6) by permutational multivariate analysis of variance (PERMANOVA) of pairwise generalized UniFrac distance matrices, with 1000 permutations.

To identify significant associations between metadata and transformed microbial taxonomic or functional abundances, we applied two multivariate regression models that allow for mixed effects (Multivariate microbial Association by Linear models, MaAsLin (Morgan et al., 2012) and Inference for Absolute Abundance, IFAA (Li et al., 2021)). MaAsLin utilizes robust additive general linear models on relative abundances and IFAA employs robust estimating equations for parameter estimation of differential absolute abundance. Both methods were able to establish associations and identify differentially abundant taxa while adjusting for multiple time points and other confounders, MaAsLin results imply direction of associations (coefficients and *q* values) with respect to relative abundance while IFAA results inferred magnitude of change in absolute abundance (estimates and 95% confidence intervals). Additional information regarding these methods can be found in Supplementary Methods.

Shotgun metagenomic reads were input into the HUMAnN2 (HMP Unified Metabolic Analysis Network) suite of tools under default parameters (Franzosa et al., 2018). MetaPhlAn2 was used to extract taxonomical profiles while functional pathways were assigned to reads based on the *chocophlan* databases and genes based on *UniRef90* (Suzek et al., 2015). The HUMAnN2 gene abundance table was regrouped and mapped based on MetaCyc database (Caspi et al., 2018). Similar to 16S sequencing data and using the same parameters, the resulting taxonomic and pathway abundance tables from HUMAnN2 were analyzed with MaAsLin and IFAA to determine significant features associated with the comparison groups within a multivariable model.

## Results

### Participant Characteristics

A total of 500 stool samples collected from 229 children from NHBCS across several time-points in the first year of life were included in this study. Infant participants comprised 55% (*n* = 127) males and 28% (*n* = 64) were CD. The maternal and infant characteristics stratified by delivery mode and feeding type at time of sample collection are shown in Table 1.

**TABLE 1:** Characteristics of the Mother-Infant Dyads by Delivery Mode (*N* = 229).

Maternal characteristics	Vaginal (*N* = 165) n (%)	Cesarean (*N* = 64) n (%)	*P*
Maternal age in years, mean (SD)	32 (4)	33 (4)	0.07
Use of antibiotics during pregnancy	68 (41)	57 (89)	**0.02**
BMI (kg/m^2^), mean (SD)	25 (5)	27 (6)	0.06
**Infant characteristics**			
Male	88 (53)	39 (61)	0.37
Gestational age in weeks, mean (SD)	39 (1)	38 (2)	**0.007**
Infant birth weight in g, mean (SD)	3440 (499)	3405 (516)	0.64
Feeding mode at 6 weeks			0.57
EBF	90 (54)	30 (47)	
Mixed feeding	55 (33)	23 (36)	
EFF	5 (3)	4 (6)	
Use of antibiotics over first year	45 (27)	17 (25)	0.35

*Bold values indicate *P* < 0.05.*

By design, majority of the samples were collected at 6 weeks and 1 year of life and, other specimens collected between this timeframe were oversampled for VD (Supplementary Table 1). Among participants that provided samples at 1 year, (*N* = 194; 85% of the study population), most (95%) initiated breastfeeding and 26% were EBF throughout the period of follow-up, (Supplementary Table 2). Shotgun sequencing was performed for 350 6-week and 1-year samples, with 150 infants providing both 6-week and 1-year samples (Frequencies of samples at each time point are shown in Supplementary Table 3).

### Characterization of the Infant Intestinal Microbiome in the First Year of Life

Bacterial richness and diversity increased over the first 12 months of life with the largest changes occurring between 4 months and 12 months (Supplementary Figures 1A,B, 2).

### Delivery Mode Impacts Fecal Microbial Colonization and Maturation Over the First Year of Life

**FIGURE 1:**
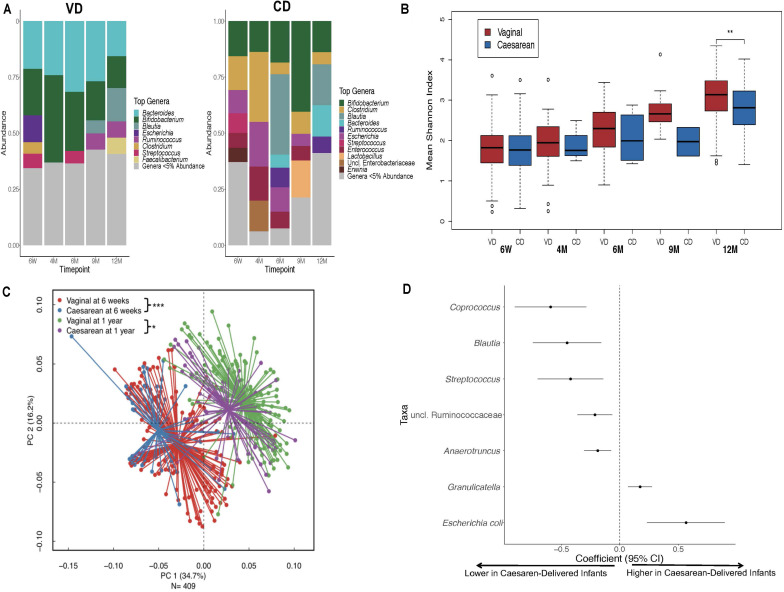
Distinct 16S microbial signatures based on birth mode in the First year of life. **(A)** Stacked bar charts illustrating relative abundance of predominant genera (≥5% relative abundance) at each period of sample collection by delivery mode. **(B)** Shannon diversity indices was significantly higher for the VD group at 1 year only, however, diversity indices were consistently lower for the CD group throughout the First year of life. **(C)** Infant stool community composition differ based on delivery mode over the course of the study period. Beta diversity, generalized Unifrac adjusted group differences between CD and VD infants met the significance threshold at 6 weeks and 1 year. **(D)** Differentially abundant taxa based on delivery mode. *p < 0.05, **p < 0.01, ***p < 0.001, NS not significant. VD, vaginally delivered; CD, cesarean -delivered.

Over the first year of life, gut colonization patterns differed by delivery mode with *Bifidobacterium* and *Bacteroides* as predominant genera in VD infants while *Bifidobacterium* and *Clostridium* were most prevalent in CD infants (Figure 1A). Samples from CD infants had consistently lower microbial diversity indices over the first 1 year of life (*P* < 0.001; Figure 1B). Generalized UniFrac group differences of beta diversity between CD and VD infants met the significance threshold at 6 weeks and 1 year (*P* < 0.001 and *P* = 0.02, respectively; Figure 1C). Clostridium difficile was higher, while Bifidobacterium and Bacteroides were in lower abundance in CD infants at baseline. Over the follow-up period, regardless of feeding mode, infants born operatively had significantly lower abundance of several taxa including Coprococcus, Blautia, and Streptococcus compared to VD infants, while *E*. *coli* and Graniculatella were enriched (Figure 1D and Supplementary Table 4).

### The Impact of Infant Feeding on the Development Infant Gut Microbiota Composition Differs by Delivery Mode

**FIGURE 2:**
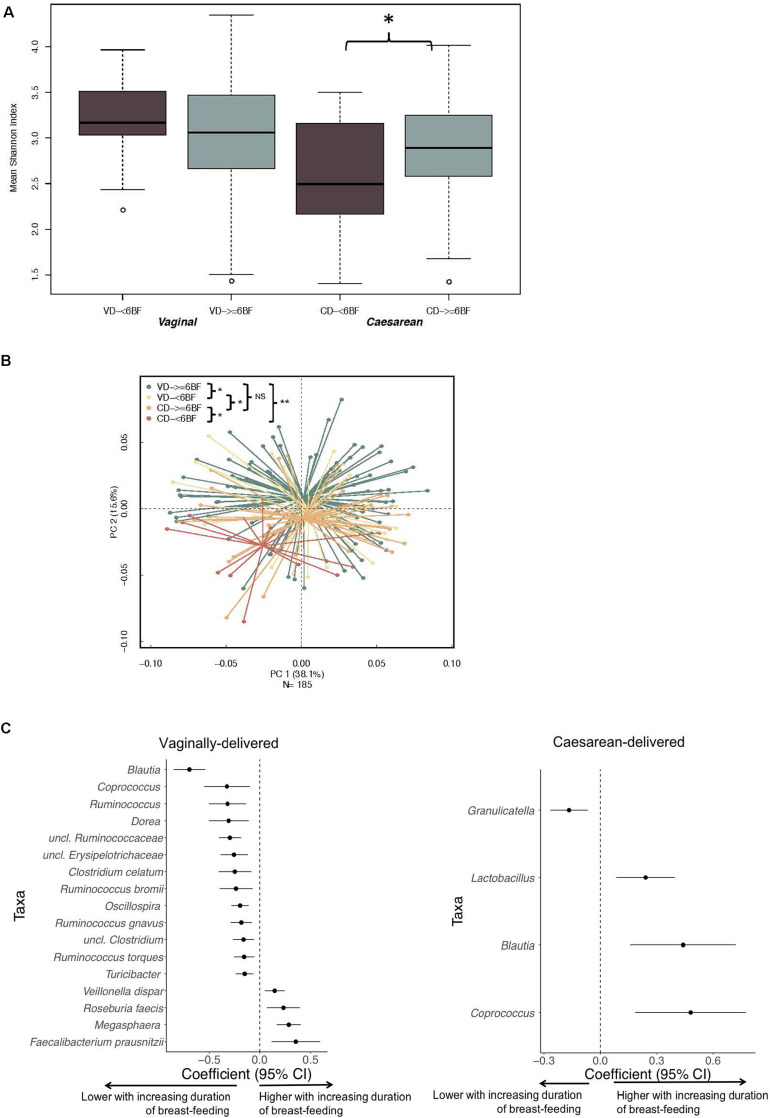
The impact of breastfeeding duration on the developing microbiota over 1 year (characterized by 16S rRNA gene) differs by delivery mode. **(A)** Breastfeeding duration was significantly associated with 1-year alpha diversity indices for CD infants and not for VD infants. **(B)** Beta diversity of gut community profiles differed by feeding mode at 6 weeks and breast-feeding duration at 1 year as visualized with PCoA plots. Adjusted *p*-values are the result of the *adonis2* model, adjusting for gestational age, early feeding mode and solid food introduction at the time of sample collection. **(C)** Differentially abundant taxa highlighting ASVs (at the genus level) significantly impacted by duration of breastfeeding for VD and CD infants adjusted for gestational age, early feeding mode and solid food introduction at the time of sample collection. Data was derived from utilizing all time-varying samples within a mixed effect model in MaAsLin. VD, vaginally delivered; CD, cesarean-delivered; VD- ≥6BF, vaginally delivered who breast-fed for >6 months; VD- <6BF, vaginally delivered who never breast-fed or breast-fed for <6 months; CD- ≥6BF, caesarean-delivered who breast-fed for ≥6 months; CD- ≥6BF, operatively delivered who never breast-fed or breast-fed for <6 months. * p < 0.05, ** p < 0.01, NS not significant.

Over the study period, FF infants consistently had lower diversity indices compared to EBF infants (multivariable analyses: 6 week, *P* = 0.05; 4 months, *P* = 0.01; 6 months, *P* = 0.03; 9 months, *P* = 0.02; 1 year, *P* = 0.09; Supplementary Figure 3A). Feeding mode distinguished 6-week and 1-year samples in PERMANOVA regression as visualized with PCoA plots (Supplementary Figure 3B). Shannon diversity for CD- ≥ 6BF was higher when compared with CD- < 6BF (*P* < 0.05), although this difference was not observed in VD infants (Figure 2A). VD- ≥ 6BF were significantly different in community composition at 1 year compared with all other comparison groups (Figure 2B). Notably, CD- ≥ 6BF did not significantly differ from VD- ≥ 6BF.

MaAsLin2 results from 16S data revealed eleven species including *Bacteroides fragilis*, *Clostridium difficile*, *Oscillospira* and *Acinetobacter*, with statistically significant differences in relative abundance (Supplementary Table 4), while five taxa including *Bifidobacterium* and *Bacteroides sp.*, were identified to differ in absolute abundance (Supplementary Table 5) when comparing CD and VD infants at baseline. Notably both methods (MaAsLin2 and IFAA) revealed that *B. fragilis* was depleted at baseline in CD infants (Supplementary Tables 4–6). MaAsLin2 revealed a negative association comparing CD to VD infants (coef. =− 0.031, standard error = 0.009, *Q* = 0.04) and IFAA results showed a 92% (95% CI: 82–97%) reduction in CD infants in terms of absolute abundance.

When examining follow-up periods, taxa associated with duration of breast-feeding were different for VD and CD infants (Figure 2C and Supplementary Table 4). 16S data revealed that for CD infants, levels of *Lactobacillus* increased with longer duration of breastfeeding (MaAsLin2 -coef. −0.032, standard error = 0.0008, *Q* = 0.01) while several other taxa including Peptostrepococcaceae sp., *Faecalibacterium prausnitzii, Turicibacter and Acinetobacter* were depleted with longer duration of breastfeeding. Three taxa (*Coprococcus, Blautia*, and *Streptococcus)* negatively associated with CD (Figure 1D), subsequently increased in abundance with increasing duration of breastfeeding (Figure 2C). Among CD infants, longer duration of breast-feeding increased the absolute abundance of *B. fragilis* by 22% (95% CI −9–48%) (Supplementary Table 5). There were no significant differences between CD and VD infants that breast-fed for more than 6 month (*Q* > 0.1; Supplementary Table 6).

**FIGURE 3:**
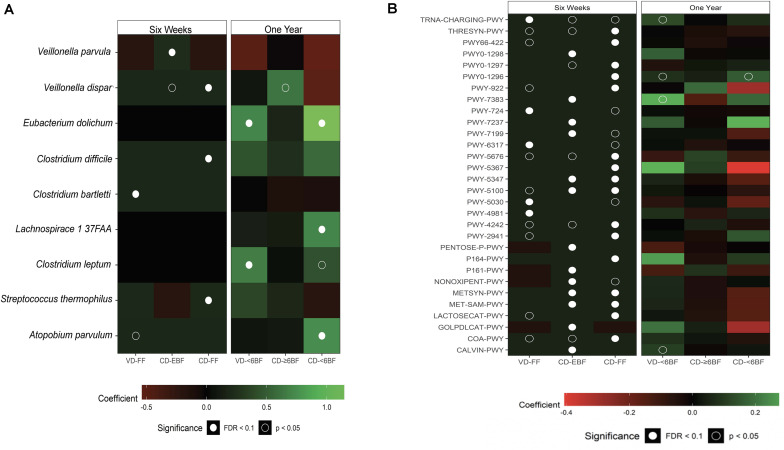
Multivariable results from metagenomic sequencing (species level taxa and MetaCyc pathways). Heat maps showing the direction and size of model coefficients as determined by MaAsLin2 modeling **(A)** significant taxonomic differences and **(B)** significant functional pathways that differed relative to VD-EBF at 6 weeks and relative to VD-≥6BF at 1 year. Figures include identified taxa with a FDR corrected *q* value < 0.25. VD-EBF, vaginally delivered and EBF; VD-FF, vaginally delivered and FF; CD-EBF, cesarean -delivered and EBF; CD-FF, cesarean -delivered who were FF; VD- ≥ 6BF, vaginally delivered who breast-fed for >6 months; VD- < 6BF, vaginally delivered who never breast-fed or breast-fed for <6 months; CD- ≥ 6BF, cesarean -delivered who breast-fed for ≥6 months; CD- ≥ 6BF, operatively delivered who never breast-fed or breast-fed for <6 months; FDR, false discovery rate. TRNA-CHARGING-PWY, tRNA charging; THRESYN-PWY, superpathway of L-threonine biosynthesis; PWY66-422, D-galactose degradation V (Leloir pathway); PWY0-1298, superpathway of pyrimidine deoxyribonucleosides degradation; PWY0-1297, superpathway of purine deoxyribonucleosides degradation; PWY0-1296, purine ribonucleosides degradation; PWY-922, mevalonate pathway I; PWY-7383, anaerobic energy metabolism; PWY-724, superpathway of L-lysine, L-threonine and L-methionine biosynthesis II; PWY-7237, myo-, chiro- and scillo-inositol degradation; PWY-7199, pyrimidine deoxyribonucleosides salvage; PWY-6317, galactose degradation I; PWY-5676, acetyl-CoA fermentation to butanoate II; PWY-5347, superpathway of L-methionine biosynthesis;PWY-5100, pyruvate fermentation to acetate and lactate II; PWY-5030, L-histidine degradation III; PWY-4981, L-proline biosynthesis II (from arginine); PWY-4242, pantothenate and coenzyme A biosynthesis III; PWY-2941, L-lysine biosynthesis II; PENTOSE-P-PWY, pentose phosphate pathway; P164-PWY, purine nucleobases degradation I; P161-PWY, acetylene degradation; NON-OXIPENT-PWY, pentose phosphate pathway; METSYN-PWY, L-homoserine and L-methionine biosynthesis; MET-SAM-PWY, superpathway of S-adenosyl-L-methionine biosynthesis; LACTOSECAT-PWY, lactose and galactose degradation I; GOLPDLCAT-PWY, superpathway of glycerol degradation to 1,3-propanediol; COA-PWY, coenzyme A biosynthesis I; CALVIN-PWY, Calvin-Benson-Bassham cycle.

To further investigate the impact of breastfeeding across groups, we examined taxonomic and functional pathway abundance using shotgun data at 6 weeks and 1 year (Figures 3A,B). All taxa associated with duration of breast feeding were in the Firmicutes phylum, with several taxa within *Clostridium* (including *C. difficile), Apotobium* and *Lachnospiraceae* being higher (Q ≤ 0.1) for all groups compared to VD- ≥ 6BF (Supplementary Table 7). The relative abundance of several pathways was associated with delivery mode and breastfeeding status at 6 weeks of life, including a few related to sugar synthesis and degradation (e.g., Calvin cycle, gluconeogenesis, lactose, and galactose degradation- Figure 3B and Supplementary Table 8). Although some pathways were nominally different from VD≥6BF in 1-year samples, none of these associations were significant after adjusting for multiple comparisons.

## Discussion

We performed a longitudinal study of 229 infants to evaluate the individual and combined effect of delivery and feeding methods on the developing gut microbiota over the first year of life. To our knowledge, this is the first study addressing the long-term combined effects of delivery and feeding within a US-based cohort. We observed statistically significant differences in the developing microbial diversity, composition and structure in CD infants compared with VD infants that persist to 1 year of life. Furthermore, we observed divergences based on feeding mode and length of breast feeding. Microbial diversity and taxonomic composition of the gut microbiota related to duration of breastfeeding differed for CD infants compared with VD infants.

The gut microbiota of infants changed extensively over the first year of life in our study. Over the first year of life, we observed an increase of microbes such as *Blautia, Ruminococcus* and *Faecalibacterium*, while bacteria such as *Bacteroides, Bifidobacterium*, *Escherichia coli, Staphylococcus* and *Klebsiella* decreased over time. Delivery mode has been found to relate to the baseline composition of the infant gut microbial community with higher levels of *Clostridium perfringens* and lower levels of *Bacteroidetes, Bacteroides fragilis* and *Bifidobacterium* in CD infants compared with VD infants (Penders et al., 2006; Madan et al., 2016; Stewart et al., 2018; Yang et al., 2019; Singh et al., 2020). Consistent with previous reports, our comparison of delivery modes conveyed a decrease in *Bacteroidetes* over time in CD infants, while *Bifidobacterium* remained the dominant genus in the VD group in this critical developmental period (Liu et al., 2019; Yang et al., 2019). Differences in early microbial colonization patterns are important, not only because of their potential impact on the final composition of the microbiota, but also because they will influence the concomitant development of the infant’s immune system. Therefore, the disrupted colonization due to C-section may be reflected in an altered development of the immune system, with potential long-term consequences.

Our data show that the longer CD infants were breast-fed, the higher their intestinal microbial diversity at 1 year, suggesting that the duration of breast-feeding has a stronger impact on the gut microbiota of CD infants as compared with VD infants. We also observed that higher levels of taxa including *Oscillospira* and *Bacteroides fragilis* (previously depleted at baseline in CD infants) were associated with longer duration of breastfeeding. *Faecalibacterium prausnitzii, Peptostreptococcaceae*, and *Acinetobacter* sp. were also observed to “reverse” colonization with breastfeeding. This modifying effect of breast-feeding might explain the partial restoration of the gut microbiota in CD infants observed by others (Dominguez-Bello et al., 2016; Hill et al., 2017; Stewart et al., 2018; Liu et al., 2019). Specifically, a study of Chinese infants (Liu et al., 2019) and the INFANTMET cohort (Hill et al., 2017) indicated that breastfeeding may restore the gut microbiota of operatively delivered infants to resemble vaginal-born breast-fed infants at 6 weeks and 24 weeks, respectively. Furthermore, Yang et al. (Yang et al., 2019) found that infant feeding mode had a more pronounced contribution toward changes in microbiota than delivery mode.

Infant feeding represents the next major early-life exposure following birth that shapes the infant gut microbiome, and human milk is an important source of nutrition and bioactive factors with multifunctional components that shape the developing immune system (Iwasaki and Medzhitov, 2015; McGuire and McGuire, 2015). Human breast milk, via its residing oligosaccharides (HMOs), serve as prebiotics, and the human milk microbiota provide early life probiotics, shaping the developing infant intestinal microbiome by supporting and facilitating the growth of beneficial microbes and developing immune functions (Newburg and He, 2015; Williams et al., 2019). Many studies (Forbes et al., 2018; Liu et al., 2019) have reported on the perturbation of the infant gut microbiota with formula supplementation. Gut microbiota of formula-fed and vaginal-born CHILD study infants were less enriched with family Veillonellaceae and Clostridiaceae compared to their breastfed, vaginally delivered counterparts (Yasmin et al., 2017). These changes were also evident in cesarean -delivered infants to a lesser extent. Liu et al. (Liu et al., 2019) reported that the relative abundances of *Enterococcus, Veillonella*, and *Faecalibacterium* were different between exclusively breast-fed and formula-fed CD infants.

Our findings with metagenomics clarified our 16S results, with more precise classification to the species level. Of note, CD infants who received formula supplementation had higher carriage of the pathogen *C. difficile* in early life, increasing the probability of symptomatic infection in the infant or other family members (Schaffler and Breitruck, 2018). At 1 year, infants who received formula supplementation before six months – especially CD infants – had more *Eubacterium dolichum*, a bacterium associated with higher visceral fat mass (Pallister et al., 2017), suggesting a mechanism by which infant diet and delivery mode impact childhood body composition. We found that the metabolic potential of the infant gut is substantially disrupted by both cesarean delivery and formula supplementation, but is restored by 1 year. Metatranscriptomic studies are needed to confirm whether similar bacterial functional capacity results in similar gene expression.

Our study is not without limitations. 16S sequencing data, which has incomplete resolution to species and strain level, limits the extent to which we can explain our findings (Janda and Abbott, 2007). While our analysis adjusted for solid food introduction, our results do not provide information about the impact of weaning and solid food intake which are critical components of infant feeding in the first year of life.

Furthermore, we only analyzed a subset of samples (∼ 25 samples each) at 4, 6, and 9 month time points, and so most of our samples were collected at 6 weeks and 1 year. We therefore cannot make inferences about the interval between 6 weeks and 1 year. Nevertheless, evaluating this subset enables examination of the trajectory of microbiota development in infancy in a birth cohort study which includes, by definition, a varied population of subjects in a time in the lifespan which is understudied with respect to the human microbiome despite its tremendous importance with respect to immune training for lifelong health outcomes. We were not able to integrate other biologics (like breast milk, urine or vaginal samples) and associated ‘omics data to further inform our results (Ursell et al., 2014; Hill et al., 2017; Kirmiz et al., 2018). However, in a previously published report from our cohort (Lundgren et al., 2019), six-week infants with detectable *Acinetobacter* in their stool had mothers with detectable *Acinetobacter* in their breast milk. While we were not able confirm that the 16S sequencing reads originate from the same strain in paired maternal milk and infant stool, it is clear that breast milk contributes to the infant gut microbiome through multiple mechanisms in addition to immune modulation by breast milk metabolites and direct colonization of the infant gut.

Epidemiological studies in humans and their associated mechanistic studies in animal models have clarified that the interaction of the developing microbiota with the developing immune system occurs in a critical window of time in early infancy (Russell et al., 2015; Livanos et al., 2016). Disrupted microbiota in the first months of life is associated with immune-mediated diseases, such as allergies, atopy, and asthma (Ling et al., 2014; Arrieta et al., 2015; Azad et al., 2015; Fujimura et al., 2016; Hand et al., 2016; Lynch and Boushey, 2016; Indrio et al., 2017; Kollmann et al., 2017; Butel et al., 2018; Felix et al., 2018; Borbet et al., 2019; Dang and Marsland, 2019; Hold and Hansen, 2019). Lower bacterial diversity and paucity of specific microbes in infancy, specifically *Bifidobacterium, Akkermansia*, and *Faecalibacterium*, high abundances of fungi, and distinct fecal metabolites were associated with higher risk of atopy and asthma (Fujimura et al., 2016). Nevertheless, additional large prospective ‘omic studies are required to evaluate changes in this window between delivery and weaning, to understand mechanisms by which the gut microbiome may be manipulated to optimize health or improve vaccine efficacy in infancy and beyond (Backhed, 2010; Martin et al., 2010; Madan et al., 2012; Simon et al., 2015; Nguyen et al., 2016).

## Conclusion

In this epidemiological investigation of the developing microbiome in early life, we have identified that delivery mode has a persistent effect on the infant microbiota up to 1 year of life. Breastfeeding, and longer duration of breast milk exposure, provide a profound impact on the developmental trajectory of the microbiome in operatively infants, signaling a corrective influence of breast milk. As the first weeks and months of life represent the most profound window of microbiota and immune development, these exposure-related differences represent important areas that can be leveraged for health-promoting interventions.

## Data Availability Statement

The sequencing data used in this study are available at NCBI Sequence Read Archive under accession number PRNJA296814. Epidemiologic data are not publicly available due to their sensitive and identifiable nature. No unique algorithms were generated for this work and where specialized packages have been implemented, citations are provided. Detailed code is available upon request from MC (mc2190@sdm.rutgers.edu or Modupe.O.Coker@dartmouth.edu). Requests to work with the New Hampshire Birth Cohort Study should be directed to MK (Margaret.R.Karagas@Dartmouth.edu).

## Ethics Statement

The studies involving human participants were reviewed and approved by Center for the Protection of Human Subjects at Geisel School of Medicine, Dartmouth provided ethic approval of this study (STUDY00020844: New Hampshire Birth Cohort Study. Written informed consent to participate in this study was provided by the participants’ legal guardian/next of kin.

## Author Contributions

MC had full access to all the data used for this study and took responsibility for the integrity and accuracy of the data analysis. JM, MC, and AH designed the study. JM, AH, and MK obtained funding for this study. EB, TP, HM, and MK carried out sample processing and data acquisition. MC, HL, AH, MH, ED, ZL, and JM carried out the analysis and interpretation of data. All authors read, corrected and approved the latest version, and accept responsibility for the manuscript.

## Conflict of Interest

The authors declare that the research was conducted in the absence of any commercial or financial relationships that could be construed as a potential conflict of interest.

## Footnotes

1 http://huttenhower.sph.harvard.edu/kneaddata

2 http://www.R-project.org
