# Supplementary material for: Infant Feeding Alters the Longitudinal Impact of Birth Mode on the Development of the Gut Microbiota in the First Year of Life
Source: Front Microbiol. 2021 Apr 7;12:642197. doi: 10.3389/fmicb.2021.642197 (PMC8059768; doi:10.3389/fmicb.2021.642197)
Supplement: Supplementary file 1 [file Data_Sheet_1.docx]

**SUPPLEMENTARY MATERIALS**

**Infant Feeding Alters the Longitudinal Impact of Birth Mode on the Development of the Gut Microbiota in the First Year of Life**

**List of Supplementary Material**

Supplementary Methods. Ascertainment of Feeding Mode at the Time of Stool Sample Collection, Preprocessing and DADA2 procedures, Methods for Identifying Differentially Abundant Taxa (for 16S and Shotgun) using MaAsLin; Methods for Identifying Differentially Abundant Taxa (for 16S and Shotgun) using IFAA

Table S1. Distribution of samples processed for 16S rRNA sequencing by delivery mode (N=500)

Table S2. Characteristics of the Infant’s Feeding Type at the Time of Unique Sample Collection (N=500 Samples)

Table S3. Characteristics of shotgun metagenomic samples by delivery mode (N=350 Samples)

Table S4. Results from longitudinal MaAsLin modelling highlighting taxa that are associated with birth mode and duration of breast feeding at time of sample collection (16S rRNA sequencing).

Table S5. Results from IFAA modelling of 16S abundance data highlighting taxa that are associated with birth mode and duration of breast feeding at time of sample collection (16S rRNA sequencing).

Table S6. Results from MaAsLin modelling of 16S abundance data highlighting taxa that are associated with categories of children based on birth mode and form or duration of breast feeding at time of sample collection

Table S7. Results from MaAsLin modelling of WGS abundance data at 6 weeks and 1 year highlighting taxa that are associated with categories of children based on birth mode and form or duration of breast feeding at time of sample collection

Table S8. Results from MaAsLin modelling of WGS pathway abundance data at 6 weeks and 1 year highlighting taxa that are associated with categories of children based on birth mode and form or duration of breast feeding at time of sample collection

Figure S1A-B. Bacterial Richness and Diversity Increased over the First Year of Life.

eFigure S2. Taxonomic composition of the gut microbiota over time during the first year of life across all 500 samples from 229 subjects.

Figure S3. Feeding mode distinguished microbiota of 6-week and 1-year samples A) Within-sample diversity (Shannon) index by Infant Feeding mode. B) Community clustering (PCoA plots) by Infant Feeding Mode. Adjusted p-values are the result of the adonis2 model, adjusting for gestational age, delivery mode and solid food introduction at the time of sample collection; EBF, exclusively breastfed; FF, formula-fed; >=6BF, breast fed for > 6 months; <6BF, breast-fed for < 6 months *** p <0.001, NS not significant.

**Supplementary Methods.**

**Ascertainment of Feeding Mode at the Time of Stool Sample Collection**

Infant feeding mode is a variable derived from the New Hampshire Birth Cohort Study demographic, behavioral and clinical data^1^. It is a function of sample age and it is inferred from University of New Hampshire telephone questionnaire data collected at 4, 8, and 12 months of age. The telephone questionnaire data contained questions related to breastfeeding and formula use based on responses at these time points. Infant stool samples were collected at approximate times between 6 weeks and 12 months and this does not necessarily coincide with timing of questionnaire responses. Therefore, we used a consistent telephone interview response with an interview age that is older than but nearest to the sample age.

Feeding mode at the time of stool sample collection was classified as 0, if unable to determine from information available at the time of the data request; 1, for exclusively breastfed (EBF), 2, exclusively formula fed (EFF) or 3, mixed fed (MF). Groups classified under 2 or 3 were further classified as formula fed (FF). During the process of data cleaning and quality assurance, we defined a censor variable as 0 when there was zero or little confidence in inferred feeding mode value and 1 when there was reasonable confidence based on the information available. Consequently, all samples with a feeding mode or censor of 0 were removed before further analysis.

**Preprocessing and DADA2 procedures**

*Cutadapt*^2^ was used to trim adapters and thereafter DADA2^3^ was implemented to filter, trim, dereplicate, merge paired reads, and remove chimeras (using the built-in filterAndTrim function of DADA2 version 1.8 with the following parameters: truncLen = c(235, 200), trimleft = c(3, 0), maxN = 0, maxEE = 5)

**Methods for Identifying Differentially Abundant Taxa (for 16S and Shotgun) using MaAsLin**

MaAsLin^4^ [<http://huttenhower.sph.harvard.edu/maaslin>] uses a variance-stabilizing arcsine square root transformation to create continuous abundance profiles that can be analyzed by regular general linear models. As samples were collected a varying times over one year, longitudinal models in MaAsLin were constructed to examine the association between breastfeeding and 16S-identified taxon relative abundance over time. For this, MaAsLin models were fit with a random effect for subject for all available samples. Fixed effects were included for peripartum antibiotic exposure, EBF at baseline, duration of BF at follow-up sample, delivery mode, the interaction between delivery mode and EBF at baseline, the interaction between delivery mode and BF duration at follow-up, the interaction between delivery mode and follow-up period, infant sex, duration of solid food consumption, the interaction between duration of solid food consumption and follow-up period, and stool sample age. Parameters on the taxa examined included minimum percentage of samples 10% and minimum percentage relative abundance 0.001%. This method resulted in effect estimates, unadjusted p values and Benjamini–Hochberg FDR p-values (q values). Furthermore, for 16S and shotgun samples, MaAsLin was used to determine significant taxa associated with each covariate within a multivariable model that included the four groups (VD-EBF, VD-FF, CD-EBF and CD-FF for 6-week samples and VD->6BF, VD-<6BF, CD->6BF, CD-<6BF for 1-year samples). The model included intrapartum antibiotic exposure, duration of solid food consumption, and exact age at stool sample collection as covariates. Models for 1-year samples also adjusted for EBF/FF at 6 weeks. VD-EBF was the reference group for 6-week samples while VD->6BF was the reference group for 1-year samples. The effect estimates, p values and q values were derived for the association between dependent and independent variables. The following parameters were applied:- minimum percentage of samples 10%, minimum percentage relative abundance 0.001%, P < 0.05, q < 0.1 – such that only taxa that had more > 0.001% in abundance for at least 10% of samples were included in the analyses.

**Methods for Identifying Differentially Abundant Taxa (for 16S and Shotgun) using IFAA**

Making inferences with relative abundance is challenging to due to the compositional structure of the microbiome. Therefore, a novel method, IFAA^5^, was used to test the associations of absolute abundances in the stool microbiota with specific phenotypes while adjusting for potential confounders. It employs mixed effects to handle correlations and robust estimating equations for parameter estimation. IFAA identifies microbial taxa associated with the covariates and then estimates the association parameters by employing an independent reference taxon. As with MaAsLin, variables included peripartum antibiotic exposure, EBF at baseline, duration of BF at follow-up sample, delivery mode, the interaction between delivery mode and EBF at baseline, the interaction between delivery mode and BF duration at follow-up, the interaction between delivery mode and follow-up period, infant sex, duration of solid food consumption, the interaction between duration of solid food consumption and follow-up period, and stool sample age.

**References**

1. Madan JC, Hoen AG, Lundgren SN, et al. Association of Cesarean Delivery and Formula Supplementation With the Intestinal Microbiome of 6-Week-Old Infants. *JAMA pediatrics.* 2016;170(3):212-219.

2. Martin M. Cutadapt removes adapter sequences from high-throughput sequencing reads. *2011.* 2011;17(1):3.

3. Callahan BJ, McMurdie PJ, Rosen MJ, Han AW, Johnson AJ, Holmes SP. DADA2: High-resolution sample inference from Illumina amplicon data. *Nat Methods.* 2016;13(7):581-583.

4. Morgan XC, Tickle TL, Sokol H, et al. Dysfunction of the intestinal microbiome in inflammatory bowel disease and treatment. *Genome Biol.* 2012;13(9):R79.

5. Li Z, Tian L, o Malley AJ, et al. IFAA: Robust association identification and Inference For Absolute Abundance in microbiome analyses. *arXiv: Applications.* 2019.

**Table S1. Distribution of samples processed for 16S rRNA sequencing by delivery mode (N=500)***

| **Characteristics** | **Vaginal (N=371)** | **Caesarean (N= 129)** |
| --- | --- | --- |
| Time of Sample Collection, no. (%) | | |
| 6 weeks; N=215* | 154 (41) | 61 (47) |
| EBF | 125 (34) | 39 (30) |
| FF | 29 (8) | 22 (17) |
| 4 months; N=32 | 29 (8) | 3 (2) |
| 6 months; N=29 | 25 (7) | 4 (3) |
| 9 months; N=30 | 28 (7) | 2 (1) |
| 1 year; N=194* |  |  |
| BF <6 months | 41(11) | 19(15) |
| BF > 6 months | 91(24) | 40(31) |
| Duration of breast-feeding in days, mean(sd) | | |
| 6 weeks; N=215 | 41.7 (11) | 42.3(28) |
| 4 months; N=32 | 118.4(30) | 124.7(4) |
| 6 months; N=29 | 172.8 (50) | 131.7(87) |
| 9 months; N=30 | 258.8(2.2) | 283.5(0.0) |
| 1 year; N=194 | 252.2(139) | 239.5(139) |

*150 infants provided samples at 6 weeks and 1 year

**Table S2. Characteristics of the Infant’s Feeding Type at the Time of Unique Sample Collection (16S rRNA sequencing; N=500 Samples)***

| **Time of Sample Collection** | **Exclusively Breastfeeding** | **Mixed feeding** | **Formula only** |
| --- | --- | --- | --- |
| 6 weeks (N=215) | 164 | 39 | 12 |
| 4 months (N=32) | 22 | 10 | 1 |
| 6 months (N=29)^¶^ | 16 | 12 | 0 |
| 9 months (N=30)^¶^ | 15 | 15 | 0 |
| 12 months (N=194)^¶^ | 43 | 132 | 9 |

*150 infants provided samples at 6 weeks and 1 year

^&^Exclusive breastfeeding is defined as having never received formula

^¶^predominantly with solid food introduction; Solid food includes rice cereal, crackers, fruit, yogurt and non-liquid foods

**Table S3. Characteristics of shotgun metagenomic samples by delivery mode (N=350 Samples)**

| **Distribution of Samples by delivery mode (WGS Sequencing; N=350)*** | | |
| --- | --- | --- |
|  | **Vaginal (N=238) n(%)** | **Caesarean (N=112)**  **n(%)** |
| Time of Sample Collection |  |  |
| 6 weeks, N=179 | 124(52) | 55(49) |
| EBF | 100(42) | 33(29) |
| FF | 24(10) | 22(20) |
| 1 year, N=171 | 114 (67) | 57(33) |
| 1 year (excluding those who never breast-fed), N=163 | 110(46) | 53(47) |
| BF <6 months | 29(12) | 14(12) |
| BF > 6 months | 81(34) | 39(35) |

WGS, Whole genome sequencing; *150 infants provided samples at 6 weeks and 1 year

**Table S4. Results from longitudinal MaAsLin modelling highlighting 16S abundant taxa (q<0.25) that are associated with birth mode and duration of breast feeding at time of sample collection.** Q values <0.1 are marked in red. Composition of taxa in red were observed to significantly change with duration of breast-feeding among CD respectively.

| **Variable** | **Feature** | **Taxon Name** | **Comparison** | **Effect Estimate** | **SD** | **p-value** | **Q-value** |
| --- | --- | --- | --- | --- | --- | --- | --- |
| csecbfl | SV107 | *Peptostreptococcaceae sp.* | BF Duration at Follow-Up Among CD | -0.0021 | 0.0006 | 0.001 | 0.064 |
| csecs | SV107 | *Peptostreptococcaceae sp.* | CD vs. VD at Follow Up | 0.0024 | 0.0007 | <0.001 | 0.021 |
| bfeed.samp | SV113 | *Blautia obeum* | BF Duration at Follow-Up Among VD | -0.0018 | 0.0005 | 0.001 | 0.049 |
| bfeed.samp | SV13 | *Faecalibacterium prausnitzii* | BF Duration at Follow-Up Among VD | 0.0088 | 0.0032 | 0.006 | 0.160 |
| csecbfl | SV13 | *Faecalibacterium prausnitzii* | BF Duration at Follow-Up Among CD | -0.0118 | 0.0041 | 0.004 | 0.124 |
| bmode | SV14 | *Enterococcus sp* | CD vs. VD at Baseline | 0.0119 | 0.0045 | 0.009 | 0.222 |
| csecbf6 | SV145 | *Lactobacillus sp.* | EBF vs. FF at Baseline Among CD | -0.0019 | 0.0006 | 0.001 | 0.064 |
| csecbfl | SV145 | *Lactobacillus sp.* | BF Duration at Follow-Up Among CD | 0.0032 | 0.0008 | <0.001 | 0.010 |
| bmode | SV1486 | *Streptococcus sp.* | CD vs. VD at Baseline | 0.0000 | 0.0000 | 0.008 | 0.210 |
| bmode | SV164 | *Ruminococcus torques* | CD vs. VD at Baseline | 0.0008 | 0.0003 | 0.001 | 0.064 |
| bfeed.samp | SV170 | *Megasphaera sp* | BF Duration at Follow-Up Among VD | 0.0012 | 0.0003 | <0.001 | 0.014 |
| bfeed.samp | SV213 | *Oscillospira sp.* | BF Duration at Follow-Up Among VD | -0.0005 | 0.0002 | 0.003 | 0.108 |
| csecbf6 | SV232 | *Clostridium difficile* | EBF vs. FF at Baseline Among CD | -0.0006 | 0.0002 | 0.009 | 0.220 |
| bmode | SV232 | *Clostridium difficile* | CD vs. VD at Baseline | 0.0014 | 0.0003 | <0.001 | <0.001 |
| csecs | SV232 | *Clostridium difficile* | CD vs. VD at Follow Up | -0.0010 | 0.0003 | 0.001 | 0.066 |
| csecbf6 | SV266 | *Haemophilus parainfluenzae* | EBF vs. FF at Baseline Among CD | 0.0004 | 0.0001 | 0.004 | 0.129 |
| csecbfl | SV275 | *Turicibacter* | BF Duration at Follow-Up Among CD | -0.0007 | 0.0002 | <0.001 | 0.004 |
| csecs | SV275 | *Turicibacter* | CD vs. VD at Follow Up | 0.0008 | 0.0002 | <0.001 | <0.001 |
| csecs | SV289 | *Streptococcus sp* | CD vs. VD at Follow Up | 0.0003 | 0.0001 | 0.011 | 0.248 |
| bmode | SV3 | *Bacteroides fragilis* | CD vs. VD at Baseline | -0.0306 | 0.0089 | 0.001 | 0.040 |
| bfeed.6W | SV4 | *Clostridium uncl.* | EBF vs. FF at Baseline Among VD | -0.0156 | 0.0054 | 0.004 | 0.124 |
| bmode | SV48 | *[Ruminococcus]* | CD vs. VD at Baseline | 0.0073 | 0.0026 | 0.005 | 0.146 |
| csecs | SV48 | *[Ruminococcus]* | CD vs. VD at Follow Up | -0.0077 | 0.0029 | 0.009 | 0.218 |
| csecbf6 | SV528 | *Acinetobacter sp* | EBF vs. FF at Baseline Among CD | 0.0004 | 0.0001 | <0.001 | 0.017 |
| csecbfl | SV528 | *Acinetobacter sp* | BF Duration at Follow-Up Among CD | -0.0006 | 0.0001 | <0.001 | 0.004 |
| csecs | SV528 | *Acinetobacter sp* | CD vs. VD at Follow Up | 0.0005 | 0.0001 | 0.001 | 0.040 |
| bfeed.6W | SV53 | *Enterococcus sp* | EBF vs. FF at Baseline Among VD | -0.0033 | 0.0011 | 0.003 | 0.108 |
| bfeed.samp | SV62 | *Ruminococcus gnavus* | BF Duration at Follow-Up Among VD | -0.0040 | 0.0012 | 0.001 | 0.046 |
| bmode | SV63 | *Clostridium uncl.* | CD vs. VD at Baseline | 0.0086 | 0.0021 | <0.001 | 0.008 |
| bfeed.samp | SV66 | *Clostridium celatum* | BF Duration at Follow-Up Among VD | -0.0031 | 0.0010 | 0.003 | 0.094 |
| csecbf6 | SV67 | *Oscillospira sp.* | EBF vs. FF at Baseline Among CD | -0.0029 | 0.0009 | 0.002 | 0.081 |
| bmode | SV67 | *Oscillospira sp.* | CD vs. VD at Baseline | 0.0035 | 0.0011 | 0.001 | 0.063 |
| bmode | SV73 | *Lactococcus sp.* | CD vs. VD at Baseline | 0.0067 | 0.0021 | 0.002 | 0.067 |
| csecbfl | SV85 | *Peptostreptococcaceae sp.* | BF Duration at Follow-Up Among CD | -0.0017 | 0.0006 | 0.007 | 0.177 |
| csecs | SV85 | *Peptostreptococcaceae sp.* | CD vs. VD at Follow Up | 0.0020 | 0.0006 | 0.002 | 0.077 |
| csecs | SV876 | *Granulicatella sp.* | CD vs. VD at Follow Up | 0.0001 | 0.0000 | 0.002 | 0.067 |
| csecbf6 | SV88 | *Dorea sp.* | EBF vs. FF at Baseline Among CD | -0.0027 | 0.0008 | 0.001 | 0.044 |
| bmode | SV88 | *Dorea sp.* | CD vs. VD at Baseline | 0.0035 | 0.0009 | <0.001 | 0.010 |
| bmode | SV9 | *Streptococcus sp.* | CD vs. VD at Baseline | 0.0134 | 0.0044 | 0.002 | 0.090 |

CD, Caesarean-delivered; VD, Vaginal-delivered; EBF, Exclusively Breast-fed; FF, Formula-Fed; BF Duration, Duration of Breast-feeding

**Table S5. Results from IFAA modelling of 16S abundance data highlighting significant taxa that are associated with birth mode and duration of breast feeding at time of sample collection.** *Bacteroides fragilis* was observed to be significantly lower in CD infants at baseline but with every unit change in breast feeding duration (per month), it’s abundance increased. The impact of breast feeding on *Faecalibacterium prausnitzii* differed for CD and VD infants. *The changes reflected by the Beta coef are on the log scale of absolute abundance. It can be converted to changes on the original scale of absolute abundance.*

| **Taxon Name** | **Comparison** | **Beta Coef.** | **Low 95%CI** | **Up 95%CI** |
| --- | --- | --- | --- | --- |
| *Faecalibacterium prausnitzii* | BF duration Among VD | 0.178082 | 0.050961 | 0.310774 |
| *Bacteroides fragilis* | BF duration Among CD | 0.203835 | 0.086403 | 0.38625 |
| *Faecalibacterium prausnitzii* | BF duration Among CD | -0.21129 | -0.39262 | -0.12656 |
| *Collinella aerofaciens* | BF duration Among CD | -0.19754 | -0.36339 | -0.04102 |
| *Bifidobacterium* | CD vs. VD at Baseline | -0.95194 | -1.7441 | -0.53545 |
| *Bacteroides fragilis* | CD vs. VD at Baseline | -2.49039 | -3.63615 | -1.71215 |
| *Bacteroides* | CD vs. VD at Baseline | -3.11914 | -4.55065 | -2.01429 |
| *Bacteroides ovatus* | CD vs. VD at Baseline | -3.22001 | -5.02833 | -2.18353 |
| *Klebsiella* | CD vs. VD at Baseline | 2.159173 | 0.642439 | 3.543188 |

CD, Caesarean-delivered; VD, Vaginal-delivered; EBF, Exclusively Breast-fed; FF, Formula-Fed; BF Duration, Duration of Breast-feeding

**Table S6. Results from MaAsLin modelling of 16S abundance data highlighting taxa (Q<0.25) that are associated with categories of children based on birth mode and form or duration of breast feeding at time of sample collection**

| **Variable** | **Feature** | **Taxon Name** | **Comparison** | **Effect Estimate** | **SD** | **p-value** | **q-value** | **Age** |
| --- | --- | --- | --- | --- | --- | --- | --- | --- |
| BFBMDUM | SV266 | *Haemophilus parainfluenzae* | CD-EBF vs. VD-EBF | 0.002 | 0.001 | 0.001 | 0.028 | Six Weeks |
| BFBMDUM | SV3 | *Bacteroides fragilis* | CD-EBF vs. VD-EBF | -0.054 | 0.021 | 0.011 | 0.246 | Six Weeks |
| BFBMDUM | SV46 | *Veillonella dispar* | CD-EBF vs. VD-EBF | 0.016 | 0.004 | 0.001 | 0.027 | Six Weeks |
| BFBMDUM | SV67 | *Oscillospira sp.* | CD-EBF vs. VD-EBF | 0.009 | 0.003 | 0.008 | 0.206 | Six Weeks |
| BFBMDUM | SV1486 | *Streptococcus sp.* | CD-FF vs. VD-EBF | 0.000 | 0.000 | 0.004 | 0.125 | Six Weeks |
| BFBMDUM | SV16 | *Blautia sp.* | CD-FF vs. VD-EBF | 0.009 | 0.003 | <0.001 | 0.027 | Six Weeks |
| BFBMDUM | SV232 | *Clostridium difficile* | CD-FF vs. VD-EBF | 0.005 | 0.001 | <0.001 | <0.001 | Six Weeks |
| BFBMDUM | SV51 | *Eubacterium dolichum* | CD-FF vs. VD-EBF | 0.018 | 0.006 | 0.002 | 0.076 | Six Weeks |
| BFBMDUM | SV63 | *Clostridium uncl.* | CD-FF vs. VD-EBF | 0.034 | 0.009 | <0.001 | 0.010 | Six Weeks |
| BFBMDUM | SV66 | *Clostridium celatum* | CD-FF vs. VD-EBF | 0.004 | 0.001 | 0.004 | 0.128 | Six Weeks |
| BFBMDUM | SV73 | *Lactococcus sp.* | CD-FF vs. VD-EBF | 0.020 | 0.007 | 0.002 | 0.076 | Six Weeks |
| BFBMDUM | SV8 | *Blautia sp.* | CD-FF vs. VD-EBF | 0.011 | 0.004 | 0.006 | 0.191 | Six Weeks |
| BFBMDUM | SV258 | *Veillonella dispar* | VD-FF vs. VD-EBF | 0.001 | 0.000 | 0.010 | 0.246 | Six Weeks |
| BFBMDUM | SV33 | *Veillonella dispar* | VD-FF vs. VD-EBF | 0.017 | 0.005 | 0.001 | 0.059 | Six Weeks |
| BFBMDUM | SV36 | *Enterococcus sp.* | VD-FF vs. VD-EBF | 0.045 | 0.010 | <0.001 | 0.002 | Six Weeks |
| BFBMDUM | SV53 | *Enterococcus sp.* | VD-FF vs. VD-EBF | 0.019 | 0.004 | <0.001 | 0.001 | Six Weeks |
| BFBMDUM | SV85 | *Peptostreptococcaceae sp.* | VD-FF vs. VD-EBF | 0.002 | 0.001 | <0.001 | 0.027 | Six Weeks |
| BFBMDUM | SV13 | *Faecalibacterium prausnitzii* | CD->6BF vs. VD->6BF | -0.973 | 0.297 | 0.001 | 0.150 | One Year |
| BFBMDUM | SV292 | *Lachnospiraceae sp.* | CD->6BF vs. VD->6BF | 0.239 | 0.070 | 0.001 | 0.150 | One Year |
| BFBMDUM | SV69 | *Faecalibacterium prausnitzii* | CD->6BF vs. VD->6BF | -0.658 | 0.217 | 0.003 | 0.215 | One Year |
| BFBMDUM | SV12 | *Bifidobacterium sp.* | CD-<6BF vs. VD->6BF | -1.439 | 0.476 | 0.003 | 0.215 | One Year |
| BFBMDUM | SV832 | *Blautia sp.* | CD-<6BF vs. VD->6BF | 0.684 | 0.203 | 0.001 | 0.150 | One Year |
| BFBMDUM | SV1135 | *Ruminococcaceae uncl.* | VD-<6BF vs. VD->6BF | 0.304 | 0.072 | <0.001 | 0.020 | One Year |
| BFBMDUM | SV127 | *Blautia sp.* | VD-<6BF vs. VD->6BF | 0.452 | 0.133 | 0.001 | 0.150 | One Year |
| BFBMDUM | SV196 | *Collinsella stercoris* | VD-<6BF vs. VD->6BF | 0.420 | 0.113 | <0.001 | 0.088 | One Year |
| BFBMDUM | SV204 | *Ruminococcus sp.* | VD-<6BF vs. VD->6BF | 0.430 | 0.131 | 0.001 | 0.150 | One Year |
| BFBMDUM | SV54 | *Blautia sp.* | VD-<6BF vs. VD->6BF | 1.143 | 0.235 | <0.001 | 0.004 | One Year |
| BFBMDUM | SV622 | *Dorea sp.* | VD-<6BF vs. VD->6BF | 0.267 | 0.085 | 0.002 | 0.176 | One Year |
| BFBMDUM | SV665 | *Defluviitalea saccharophila* | VD-<6BF vs. VD->6BF | 0.284 | 0.088 | 0.001 | 0.150 | One Year |
| BFBMDUM | *SV832* | *Blautia sp.* | VD-<6BF vs. VD->6BF | 0.675 | 0.146 | <0.001 | 0.006 | One Year |

VD-EBF, Vaginally-delivered and Exclusively breast-fed; VD-FF, Vaginally-delivered and Formula-fed; CD-EBF, Caesarean-delivered and Exclusively breast-fed; CD-FF, Caesarean-delivered and Formula-fed; VD->6BF, Vaginally-delivered and breast-fed for more than 6 months; VD-<6BF, Vaginally-delivered and breast-fed for less than 6 months; CD->6BF, Caesarean-delivered and breast-fed for more than 6 months; CD-<6BF, Caesarean-delivered and breast-fed for less than 6 months;

**Table S7. Results from MaAsLin modelling of WGS abundance data at 6 weeks and 1 year highlighting taxa that are associated (Q<0.25) with categories of children based on birth mode and form or duration of breast feeding at time of sample collection**

| **Variable** | **Feature** | **Taxon Name** | **Comparison** | **Effect Estimate** | **SD** | **p-value** | **q-value** | **Age** |
| --- | --- | --- | --- | --- | --- | --- | --- | --- |
| BFBMDUM | Taxon137 | *Veillonella dispar* | CD-FF vs. VD-EBF | 0.005 | 0.001 | 2.71E-05 | 6.51E-03 | Six Weeks |
| BFBMDUM | Taxon197 | *Clostridium difficile* | CD-FF vs. VD-EBF | 0.004 | 8E-04 | 2.86E-05 | 6.51E-03 | Six Weeks |
| BFBMDUM | Taxon34 | *Clostridium bartlettii* | VD-FF vs. VD-EBF | 0.003 | 7E-04 | 1.66E-04 | 2.53E-02 | Six Weeks |
| BFBMDUM | Taxon12 | *Streptococcus thermophilus* | CD-FF vs. VD-EBF | 0.012 | 0.004 | 9.63E-04 | 7.32E-02 | Six Weeks |
| BFBMDUM | Taxon133 | *Veillonella parvula* | CD-EBF vs. VD-EBF | 0.029 | 0.008 | 9.22E-04 | 7.32E-02 | Six Weeks |
| BFBMDUM | Taxon180 | *Anaerococcus obesiensis* | CD-FF vs. VD-EBF | 2E-04 | 7E-05 | 2.16E-03 | 1.09E-01 | Six Weeks |
| BFBMDUM | Taxon30 | *Enterococcus casseliflavus* | VD-FF vs. VD-EBF | 0.024 | 0.008 | 2.49E-03 | 1.14E-01 | Six Weeks |
| BFBMDUM | Taxon118 | *Eubacterium dolichum* | CD-<6BF vs. VD->6BF | 1.270 | 0.245 | 7.27E-07 | 8.24E-04 | One Year |
| BFBMDUM | Taxon118 | *Eubacterium dolichum* | VD-<6BF vs. VD->6BF | 0.708 | 0.177 | 1.00E-04 | 2.85E-02 | One Year |
| BFBMDUM | Taxon139 | *Lachnospiraceae bacterium 6_1_63_FAA* | CD-<6BF vs. VD->6BF | 0.760 | 0.236 | 1.58E-03 | 1.69E-01 | One Year |
| BFBMDUM | Taxon189 | *Clostridium leptum* | CD-<6BF vs. VD->6BF | 0.668 | 0.142 | 6.03E-06 | 3.42E-03 | One Year |
| BFBMDUM | Taxon196 | *Lachnospiraceae bacterium 9_1_43BFAA* | CD-<6BF vs. VD->6BF | 1.273 | 0.416 | 2.65E-03 | 2.27E-01 | One Year |
| BFBMDUM | Taxon200 | *Lachnospiraceae bacterium 4_1_37FAA* | CD-<6BF vs. VD->6BF | 0.706 | 0.197 | 4.74E-04 | 8.96E-02 | One Year |
| BFBMDUM | Taxon265 | *Atopobium parvulum* | CD-<6BF vs. VD->6BF | 0.745 | 0.197 | 2.22E-04 | 5.04E-02 | One Year |

VD-EBF, Vaginally-delivered and Exclusively breast-fed; VD-FF, Vaginally-delivered and Formula-fed; CD-EBF, Caesarean-delivered and Exclusively breast-fed; CD-FF, Caesarean-delivered and Formula-fed; VD->6BF, Vaginally-delivered and breast-fed for more than 6 months; VD-<6BF, Vaginally-delivered and breast-fed for less than 6 months; CD->6BF, Caesarean-delivered and breast-fed for more than 6 months; CD-<6BF, Caesarean-delivered and breast-fed for less than 6 months

**Table S8. Results from MaAsLin modelling of WGS abundance data at 6 weeks and 1 year highlighting taxa that are associated (Q<0.25) with categories of children based on birth mode and form or duration of breast feeding at time of sample collection**

| Variable | Feature | Pathway | Level | Effect Estimate | SD | P value | Q value | Age |
| --- | --- | --- | --- | --- | --- | --- | --- | --- |
| BFBMDUM | ARG+POLYAMINE-SYN | ARG+POLYAMINE-SYN: superpathway of arginine and polyamine biosynthesis | CD-EBF vs. VD-EBF | 3.42E-05 | 1.27E-05 | 7.709E-03 | 2.250E-01 | Six Weeks |
| BFBMDUM | CALVIN-PWY | CALVIN-PWY: Calvin-Benson-Bassham cycle | CD-EBF vs. VD-EBF | 8.87E-05 | 2.54E-05 | 6.320E-04 | 6.800E-02 | Six Weeks |
| BFBMDUM | GLUCONEO-PWY | GLUCONEO-PWY: gluconeogenesis I | CD-EBF vs. VD-EBF | 4.17E-05 | 1.56E-05 | 8.133E-03 | 2.303E-01 | Six Weeks |
| BFBMDUM | GOLPDLCAT-PWY | GOLPDLCAT-PWY: superpathway of glycerol degradation to 1,3-propanediol | CD-EBF vs. VD-EBF | 3.31E-05 | 8.51E-06 | 1.439E-04 | 3.080E-02 | Six Weeks |
| BFBMDUM | HOMOSER-METSYN-PWY | HOMOSER-METSYN-PWY: L-methionine biosynthesis I | CD-EBF vs. VD-EBF | 4.95E-05 | 1.80E-05 | 6.677E-03 | 2.074E-01 | Six Weeks |
| BFBMDUM | MET-SAM-PWY | MET-SAM-PWY: superpathway of S-adenosyl-L-methionine biosynthesis | CD-EBF vs. VD-EBF | 6.63E-05 | 2.09E-05 | 1.785E-03 | 9.448E-02 | Six Weeks |
| BFBMDUM | METSYN-PWY | METSYN-PWY: L-homoserine and L-methionine biosynthesis | CD-EBF vs. VD-EBF | 6.75E-05 | 2.06E-05 | 1.245E-03 | 8.266E-02 | Six Weeks |
| BFBMDUM | NONOXIPENT-PWY | NONOXIPENT-PWY: pentose phosphate pathway (non-oxidative branch) | CD-EBF vs. VD-EBF | 1.15E-04 | 3.46E-05 | 1.069E-03 | 7.916E-02 | Six Weeks |
| BFBMDUM | P161-PWY | P161-PWY: acetylene degradation | CD-EBF vs. VD-EBF | 5.72E-05 | 1.58E-05 | 4.053E-04 | 6.005E-02 | Six Weeks |
| BFBMDUM | PENTOSE-P-PWY | PENTOSE-P-PWY: pentose phosphate pathway | CD-EBF vs. VD-EBF | 5.78E-05 | 1.63E-05 | 5.195E-04 | 6.800E-02 | Six Weeks |
| BFBMDUM | POLYAMSYN-PWY | POLYAMSYN-PWY: superpathway of polyamine biosynthesis I | CD-EBF vs. VD-EBF | 3.21E-05 | 1.21E-05 | 8.697E-03 | 2.428E-01 | Six Weeks |
| BFBMDUM | PPGPPMET-PWY | PPGPPMET-PWY: ppGpp biosynthesis | CD-EBF vs. VD-EBF | 3.27E-05 | 1.18E-05 | 6.139E-03 | 1.971E-01 | Six Weeks |
| BFBMDUM | PWY0-1298 | PWY0-1298: superpathway of pyrimidine deoxyribonucleosides degradation | CD-EBF vs. VD-EBF | 4.28E-05 | 1.30E-05 | 1.224E-03 | 8.266E-02 | Six Weeks |
| BFBMDUM | PWY-5100 | PWY-5100: pyruvate fermentation to acetate and lactate II | CD-EBF vs. VD-EBF | 8.76E-05 | 2.23E-05 | 1.248E-04 | 3.080E-02 | Six Weeks |
| BFBMDUM | PWY-5188 | PWY-5188: tetrapyrrole biosynthesis I (from glutamate) | CD-EBF vs. VD-EBF | 5.65E-05 | 1.87E-05 | 2.917E-03 | 1.221E-01 | Six Weeks |
| BFBMDUM | PWY-5347 | PWY-5347: superpathway of L-methionine biosynthesis (transsulfuration) | CD-EBF vs. VD-EBF | 7.16E-05 | 2.09E-05 | 7.687E-04 | 6.800E-02 | Six Weeks |
| BFBMDUM | PWY-6168 | PWY-6168: flavin biosynthesis III (fungi) | CD-EBF vs. VD-EBF | -4.67E-05 | 1.62E-05 | 4.424E-03 | 1.591E-01 | Six Weeks |
| BFBMDUM | PWY-6353 | PWY-6353: purine nucleotides degradation II (aerobic) | CD-EBF vs. VD-EBF | -5.35E-05 | 1.81E-05 | 3.615E-03 | 1.427E-01 | Six Weeks |
| BFBMDUM | PWY-6531 | PWY-6531: mannitol cycle | CD-EBF vs. VD-EBF | 9.58E-06 | 3.25E-06 | 3.631E-03 | 1.427E-01 | Six Weeks |
| BFBMDUM | PWY-6895 | PWY-6895: superpathway of thiamin diphosphate biosynthesis II | CD-EBF vs. VD-EBF | -3.37E-05 | 1.22E-05 | 6.423E-03 | 2.028E-01 | Six Weeks |
| BFBMDUM | PWY-6901 | PWY-6901: superpathway of glucose and xylose degradation | CD-EBF vs. VD-EBF | 4.28E-05 | 1.47E-05 | 4.079E-03 | 1.540E-01 | Six Weeks |
| BFBMDUM | PWY-7187 | PWY-7187: pyrimidine deoxyribonucleotides de novo biosynthesis II | CD-EBF vs. VD-EBF | 4.79E-05 | 1.77E-05 | 7.588E-03 | 2.248E-01 | Six Weeks |
| BFBMDUM | PWY-7199 | PWY-7199: pyrimidine deoxyribonucleosides salvage | CD-EBF vs. VD-EBF | 6.82E-05 | 2.07E-05 | 1.222E-03 | 8.266E-02 | Six Weeks |
| BFBMDUM | PWY-7237 | PWY-7237: myo-, chiro- and scillo-inositol degradation | CD-EBF vs. VD-EBF | 8.24E-05 | 2.28E-05 | 4.017E-04 | 6.005E-02 | Six Weeks |
| BFBMDUM | PWY-7383 | PWY-7383: anaerobic energy metabolism (invertebrates, cytosol) | CD-EBF vs. VD-EBF | 2.06E-05 | 6.46E-06 | 1.737E-03 | 9.448E-02 | Six Weeks |
| BFBMDUM | COA-PWY | COA-PWY: coenzyme A biosynthesis I | CD-FF vs. VD-EBF | 1.36E-04 | 3.61E-05 | 2.381E-04 | 4.585E-02 | Six Weeks |
| BFBMDUM | LACTOSECAT-PWY | LACTOSECAT-PWY: lactose and galactose degradation I | CD-FF vs. VD-EBF | 1.78E-04 | 4.53E-05 | 1.279E-04 | 3.080E-02 | Six Weeks |
| BFBMDUM | MET-SAM-PWY | MET-SAM-PWY: superpathway of S-adenosyl-L-methionine biosynthesis | CD-FF vs. VD-EBF | 8.91E-05 | 2.57E-05 | 6.858E-04 | 6.800E-02 | Six Weeks |
| BFBMDUM | METSYN-PWY | METSYN-PWY: L-homoserine and L-methionine biosynthesis | CD-FF vs. VD-EBF | 8.75E-05 | 2.53E-05 | 7.079E-04 | 6.800E-02 | Six Weeks |
| BFBMDUM | P164-PWY | P164-PWY: purine nucleobases degradation I (anaerobic) | CD-FF vs. VD-EBF | 2.63E-05 | 4.84E-06 | 2.093E-07 | 3.191E-04 | Six Weeks |
| BFBMDUM | PWY0-1296 | PWY0-1296: purine ribonucleosides degradation | CD-FF vs. VD-EBF | 1.49E-04 | 4.71E-05 | 1.921E-03 | 9.488E-02 | Six Weeks |
| BFBMDUM | PWY0-1297 | PWY0-1297: superpathway of purine deoxyribonucleosides degradation | CD-FF vs. VD-EBF | 1.37E-04 | 3.50E-05 | 1.285E-04 | 3.080E-02 | Six Weeks |
| BFBMDUM | PWY-2941 | PWY-2941: L-lysine biosynthesis II | CD-FF vs. VD-EBF | 1.55E-04 | 3.41E-05 | 1.063E-05 | 5.119E-03 | Six Weeks |
| BFBMDUM | PWY-4242 | PWY-4242: pantothenate and coenzyme A biosynthesis III | CD-FF vs. VD-EBF | 6.65E-05 | 2.09E-05 | 1.769E-03 | 9.448E-02 | Six Weeks |
| BFBMDUM | PWY4LZ-257 | PWY4LZ-257: superpathway of fermentation (Chlamydomonas reinhardtii) | CD-FF vs. VD-EBF | 1.16E-05 | 3.97E-06 | 4.042E-03 | 1.540E-01 | Six Weeks |
| BFBMDUM | PWY-5030 | PWY-5030: L-histidine degradation III | CD-FF vs. VD-EBF | 8.22E-06 | 2.85E-06 | 4.505E-03 | 1.591E-01 | Six Weeks |
| BFBMDUM | PWY-5100 | PWY-5100: pyruvate fermentation to acetate and lactate II | CD-FF vs. VD-EBF | 1.46E-04 | 2.75E-05 | 3.313E-07 | 3.191E-04 | Six Weeks |
| BFBMDUM | PWY-5347 | PWY-5347: superpathway of L-methionine biosynthesis (transsulfuration) | CD-FF vs. VD-EBF | 8.82E-05 | 2.57E-05 | 7.767E-04 | 6.800E-02 | Six Weeks |
| BFBMDUM | PWY-5367 | PWY-5367: petroselinate biosynthesis | CD-FF vs. VD-EBF | 5.05E-05 | 1.59E-05 | 1.815E-03 | 9.448E-02 | Six Weeks |
| BFBMDUM | PWY-5676 | PWY-5676: acetyl-CoA fermentation to butanoate II | CD-FF vs. VD-EBF | 2.55E-05 | 7.26E-06 | 5.686E-04 | 6.800E-02 | Six Weeks |
| BFBMDUM | PWY-6317 | PWY-6317: galactose degradation I (Leloir pathway) | CD-FF vs. VD-EBF | 6.79E-05 | 2.41E-05 | 5.337E-03 | 1.772E-01 | Six Weeks |
| BFBMDUM | PWY66-422 | PWY66-422: D-galactose degradation V (Leloir pathway) | CD-FF vs. VD-EBF | 8.14E-05 | 2.49E-05 | 1.294E-03 | 8.306E-02 | Six Weeks |
| BFBMDUM | PWY-7199 | PWY-7199: pyrimidine deoxyribonucleosides salvage | CD-FF vs. VD-EBF | 7.35E-05 | 2.55E-05 | 4.545E-03 | 1.591E-01 | Six Weeks |
| BFBMDUM | PWY-7392 | PWY-7392: taxadiene biosynthesis (engineered) | CD-FF vs. VD-EBF | 2.66E-05 | 9.34E-06 | 4.967E-03 | 1.708E-01 | Six Weeks |
| BFBMDUM | PWY-922 | PWY-922: mevalonate pathway I | CD-FF vs. VD-EBF | 1.32E-05 | 4.14E-06 | 1.718E-03 | 9.448E-02 | Six Weeks |
| BFBMDUM | SER-GLYSYN-PWY | SER-GLYSYN-PWY: superpathway of L-serine and glycine biosynthesis I | CD-FF vs. VD-EBF | 7.70E-05 | 2.49E-05 | 2.287E-03 | 1.074E-01 | Six Weeks |
| BFBMDUM | THRESYN-PWY | THRESYN-PWY: superpathway of L-threonine biosynthesis | CD-FF vs. VD-EBF | 1.09E-04 | 3.35E-05 | 1.423E-03 | 8.842E-02 | Six Weeks |
| BFBMDUM | LACTOSECAT-PWY | LACTOSECAT-PWY: lactose and galactose degradation I | VD-FF vs. VD-EBF | 1.32E-04 | 4.21E-05 | 2.115E-03 | 1.019E-01 | Six Weeks |
| BFBMDUM | OANTIGEN-PWY | OANTIGEN-PWY: O-antigen building blocks biosynthesis (E. coli) | VD-FF vs. VD-EBF | 6.62E-05 | 2.37E-05 | 5.934E-03 | 1.937E-01 | Six Weeks |
| BFBMDUM | P125-PWY | P125-PWY: superpathway of (R,R)-butanediol biosynthesis | VD-FF vs. VD-EBF | 2.55E-05 | 8.31E-06 | 2.505E-03 | 1.149E-01 | Six Weeks |
| BFBMDUM | PWY-4981 | PWY-4981: L-proline biosynthesis II (from arginine) | VD-FF vs. VD-EBF | 1.00E-04 | 2.15E-05 | 6.421E-06 | 4.122E-03 | Six Weeks |
| BFBMDUM | PWY-5030 | PWY-5030: L-histidine degradation III | VD-FF vs. VD-EBF | 9.21E-06 | 2.66E-06 | 6.722E-04 | 6.800E-02 | Six Weeks |
| BFBMDUM | PWY-6317 | PWY-6317: galactose degradation I (Leloir pathway) | VD-FF vs. VD-EBF | 7.47E-05 | 2.24E-05 | 1.049E-03 | 7.916E-02 | Six Weeks |
| BFBMDUM | PWY-6595 | PWY-6595: superpathway of guanosine nucleotides degradation (plants) | VD-FF vs. VD-EBF | 1.84E-05 | 6.86E-06 | 8.126E-03 | 2.303E-01 | Six Weeks |
| BFBMDUM | PWY66-422 | PWY66-422: D-galactose degradation V (Leloir pathway) | VD-FF vs. VD-EBF | 6.71E-05 | 2.31E-05 | 4.209E-03 | 1.559E-01 | Six Weeks |
| BFBMDUM | PWY-724 | PWY-724: superpathway of L-lysine, L-threonine and L-methionine biosynthesis II | VD-FF vs. VD-EBF | 8.51E-05 | 2.70E-05 | 1.913E-03 | 9.488E-02 | Six Weeks |
| BFBMDUM | TRNA-CHARGING-PWY | TRNA-CHARGING-PWY: tRNA charging | VD-FF vs. VD-EBF | 8.54E-05 | 2.00E-05 | 3.221E-05 | 1.241E-02 | Six Weeks |
| BFBMDUM | ASPASN-PWY | ASPASN-PWY: superpathway of L-aspartate and L-asparagine biosynthesis | CD-<6BF vs. VD->6BF | -3.01E-01 | 9.03E-02 | 1.075E-03 | 1.922E-01 | One Year |
| BFBMDUM | PWY-5188 | PWY-5188: tetrapyrrole biosynthesis I (from glutamate) | CD-<6BF vs. VD->6BF | 2.29E-01 | 6.86E-02 | 1.050E-03 | 1.922E-01 | One Year |
| BFBMDUM | PWY-5695 | PWY-5695: urate biosynthesis/inosine 5'-phosphate degradation | CD-<6BF vs. VD->6BF | -2.40E-01 | 6.26E-02 | 1.887E-04 | 1.805E-01 | One Year |
| BFBMDUM | PWY-5973 | PWY-5973: cis-vaccenate biosynthesis | CD-<6BF vs. VD->6BF | -2.01E-01 | 6.01E-02 | 1.058E-03 | 1.922E-01 | One Year |
| BFBMDUM | X1CMET2-PWY | 1CMET2-PWY: N10-formyl-tetrahydrofolate biosynthesis | CD-<6BF vs. VD->6BF | -1.53E-01 | 4.39E-02 | 6.683E-04 | 1.922E-01 | One Year |
| BFBMDUM | PWY-6630 | PWY-6630: superpathway of L-tyrosine biosynthesis | VD-<6BF vs. VD->6BF | -5.55E-01 | 1.63E-01 | 8.451E-04 | 1.922E-01 | One Year |
| BFBMDUM | RIBOSYN2-PWY | RIBOSYN2-PWY: flavin biosynthesis I (bacteria and plants) | VD-<6BF vs. VD->6BF | -5.52E-01 | 1.66E-01 | 1.098E-03 | 1.922E-01 | One Year |
| BFBMDUM | TRNA-CHARGING-PWY | TRNA-CHARGING-PWY: tRNA charging | VD-<6BF vs. VD->6BF | 1.32E-01 | 4.13E-02 | 1.662E-03 | 2.493E-01 | One Year |

**Figure S1A-B. Bacterial richness and diversity increased over the first year of life.** The largest changes in richness and diversity occurred between 4 to 1 year of age. Distribution of within-sample diversity over the first year of life by **(A)** delivery mode **(B)** feeding mode. Fitted lines depict the mean index with a 95% prediction band around it.

**B**


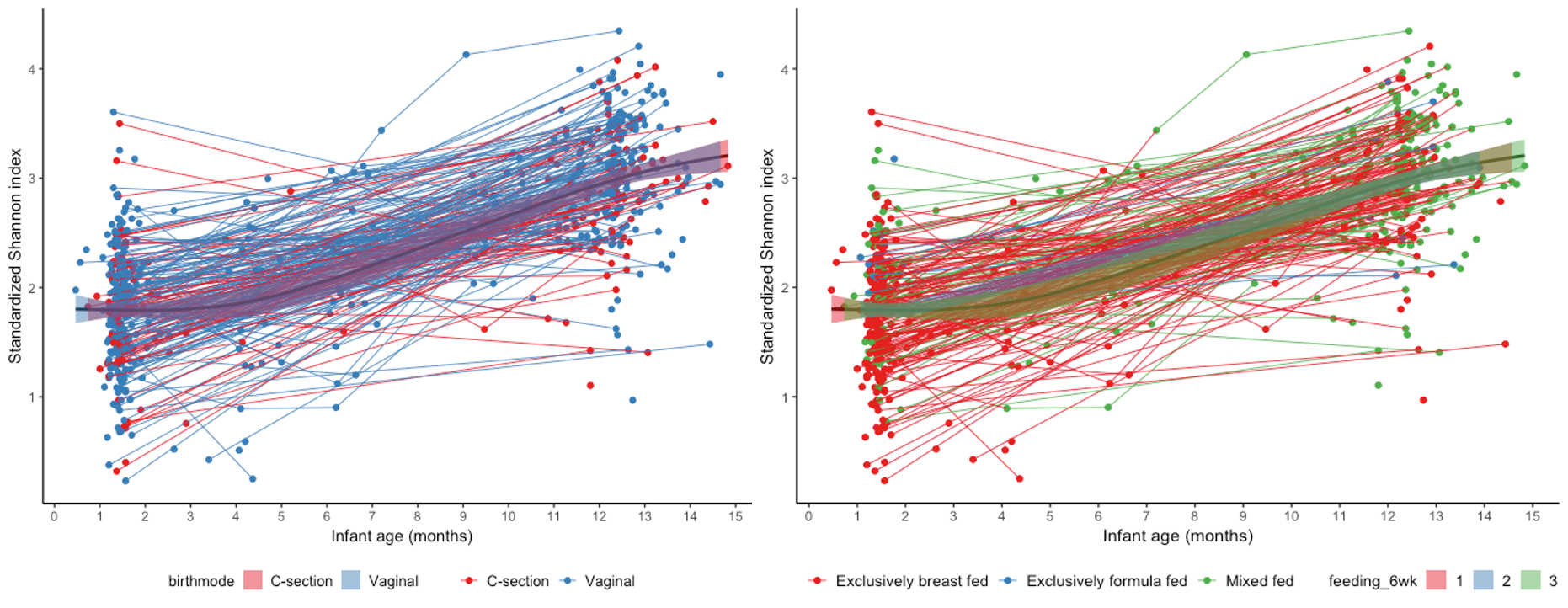


**A**

**Figure S2. Taxonomic composition of the gut microbiota over time during the first year of life across all 500 samples from 229 subjects.**

**Figure S3. Feeding mode distinguished microbiota of 6-week and 1-year samples.** Feeding mode and duration has a significant impact on gut microbiota maturation. **(A)** Within-samples diversity (Shannon) index by Infant Feeding mode. FF infants had consistently and significantly lower diversity indices compared to samples from EBF infants. **(B)** Community clustering (PCoA plots) by Infant Feeding Mode


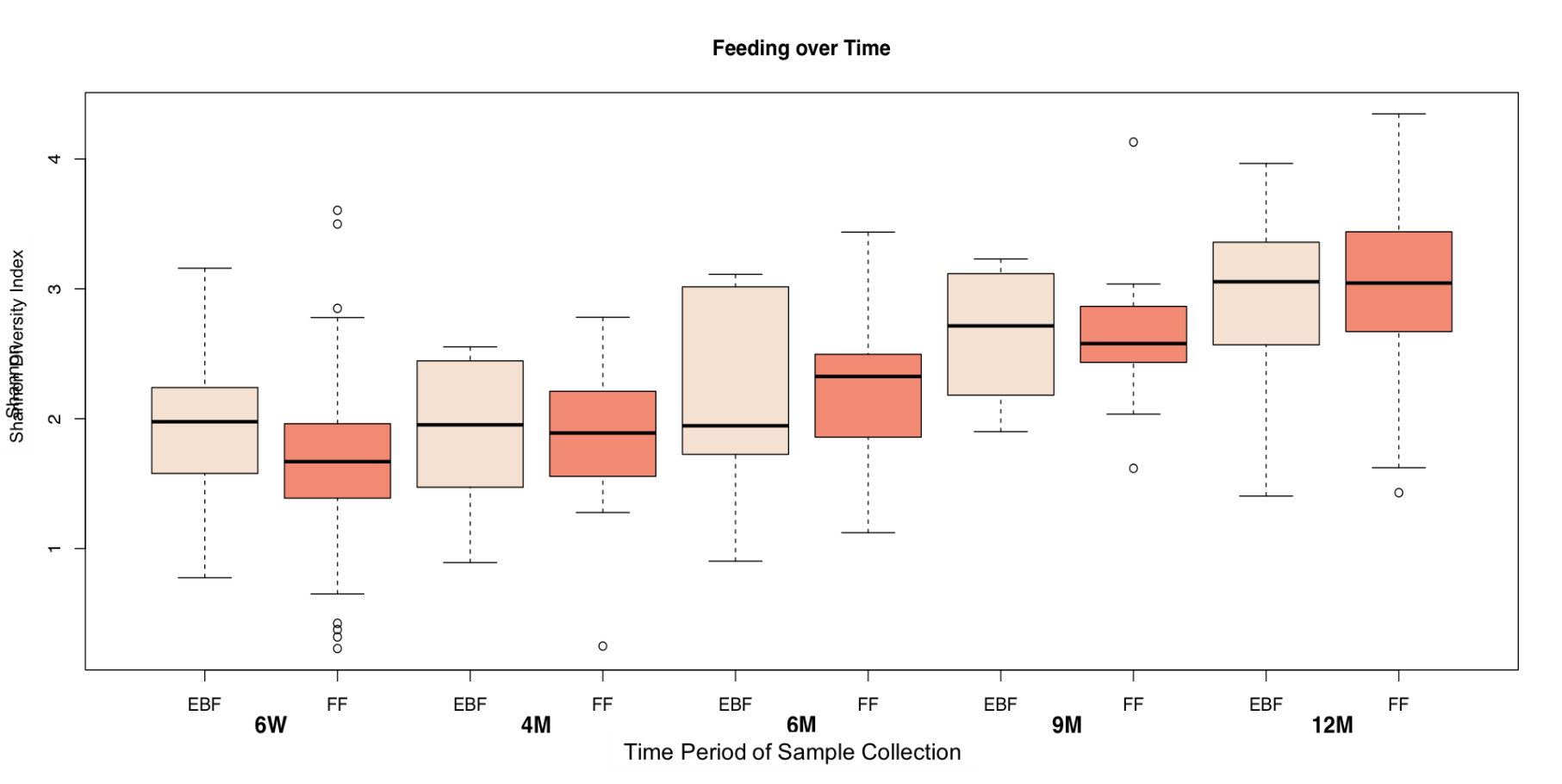


**A**

**B**
